# Supplementary material for: Modulation of anti-tumor immunity by the brain’s reward system
Source: Nat Commun. 2018 Jul 13;9:2723. doi: 10.1038/s41467-018-05283-5 (PMC6045610; doi:10.1038/s41467-018-05283-5)
Supplement: Supplementary file 1 — Supplementary Information [file 41467_2018_5283_MOESM1_ESM.pdf]

Modulation of anti-tumor immunity by the brain's reward system

Ben-Shaanan, Schiller et al.

Supplementary Figure legends:

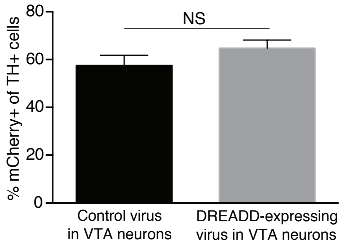

Supplementary Fig. 1

Efficiency of viral expression in the VTA dopaminergic neurons. Immunohistochemical analysis represented as percentage of mCherry positive cells of all TH^+ cells in the VTA of mice stereotactically injected with a virus carrying a gene encoding the DREADD receptor, or a control virus (encoding only the expression of the fluorescent reporter, mCherry). (*P<0.26*; Student's *t-test*; mean±s.e.m; n= 3).

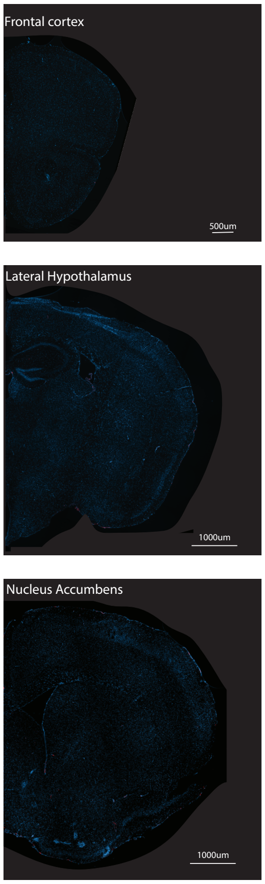

Supplementary Fig. 2

Lack of mCherry expression in the Nucleus Accumbens, Lateral Hypothalamus and Frontal Cortex. Representative images taken from mice stereotactically injected with virus in the VTA region, showing lack of neuronal expression of the fluorescent reporter mCherry in the Frontal Cortex, Lateral Hypothalamus and Nucleus Accumbens. DAPI staining is provided for visualization (mCherry- red; Dapi- blue).

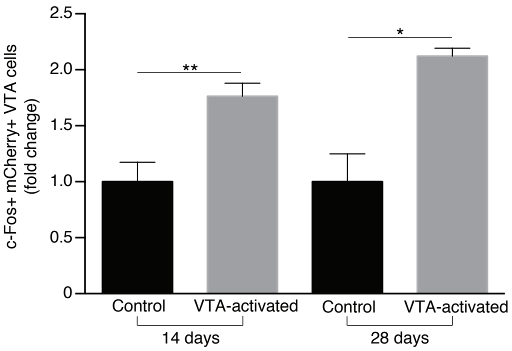

Supplementary Fig. 3

DREADD manipulation increases percentage of c-Fos positive neurons in the VTA. Quantitative immunohistochemical analysis of the percent of c-Fos expressing cells among DREADD-expressing VTA neurons following 14 or 28 days of CNO injections (for 14 days $P<0.005$, for 28 days $P<0.012$; Student's *t*-test; mean±s.e.m; n= 6, 3). Data are presented as mean fold change in c-Fos expression in mCherry positive neurons relative to the controls expressing the sham virus, and treated with CNO.

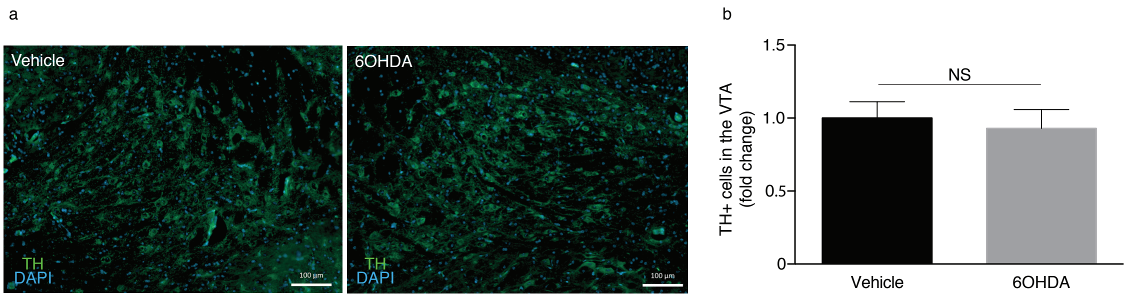

Supplementary Fig. 4

6OHDA treatment does not affect the number of TH⁺ neurons in the VTA of tumor-bearing mice (a) Representative immunohistochemical staining image of the VTA region from mice treated with 6OHDA and vehicle, stained for Tyrosine Hydroxylase (TH; green) and DAPI (blue). (b) Quantitative immunohistochemical analysis of the number of TH^+ cells in the same VTA area (mm^2) of mice treated with 6OHDA versus vehicle ($p<0.69$; Student's *t-test*; NS- not significant; n=4). Data is presented as mean fold change relative to the control group average ± s.e.m.

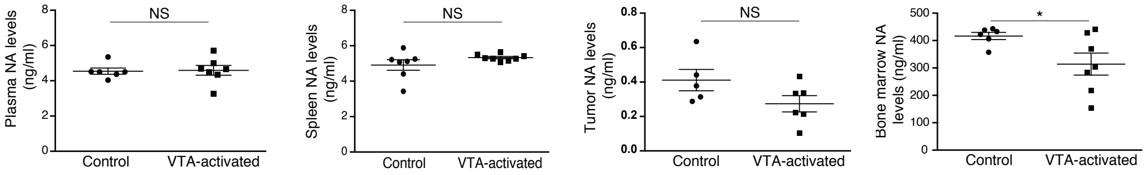

Supplementary Fig. 5

The effects of VTA-activation on noradrenaline levels in the plasma, spleen, tumor and bone marrow. Quantitative ELISA analysis of noradrenaline levels in the plasma, spleen, tumor and bone marrow of tumor-bearing mice, following daily VTA activation for 14 days. Controls were injected with a control virus and treated with CNO. (for plasma $P<0.893$ and n=6,7; spleen $P<0.16$ and n=7,8; tumor $P<0.107$ and n=5,6; bone marrow $P<0.044$ and n=6,7; Student's *t-test*; mean±s.e.m; NS-not significant). Data represent two independent repeats

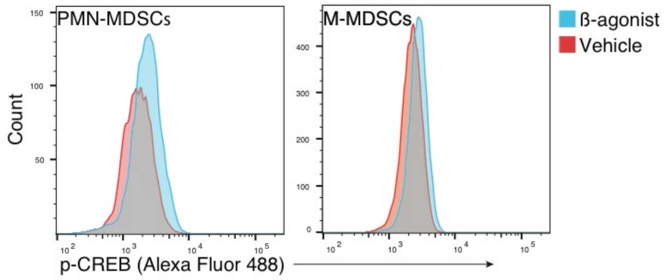

Supplementary Fig. 6

Representative histograms of p-CREB staining in bone marrow MDSCs. The populations shown in the histograms are PMN-MDSCs (CD11b$^+$ Gr-1$^+$ Ly6G$^+$) and M-MDSCs (CD11b$^+$ Gr-1$^+$ Ly6C$^+$). The X axis represents p-CREB levels.

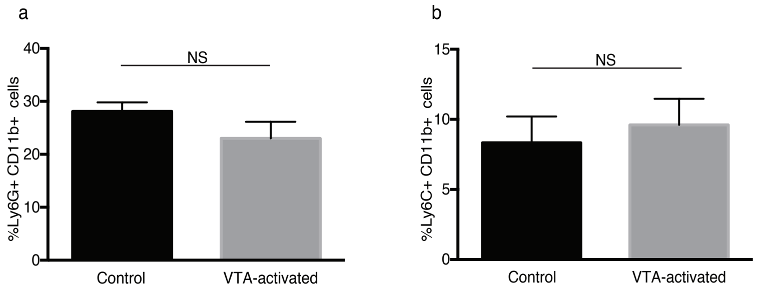

Supplementary Fig. 7

VTA activation does not affect the relative abundance of tumor PMN-MDSCs and M-MDSCs. Flow cytometry analysis of (a) PMN-MDSCs (Gr-1$^+$ CD11b$^+$ LY6G$^+$) and (b) M-MDSCs (Gr-1$^+$ CD11b$^+$ LY6C$^+$) abundance out of all tumor cells from mice subjected to daily VTA activation for 14 days, and their controls. (PMN-MDSCs $P<0.22$, n=6, 8; M-MDSCs $P<0.64$, n=6, 7; Student's *t-test*; NS-not significant). Data represent two independent repeats.

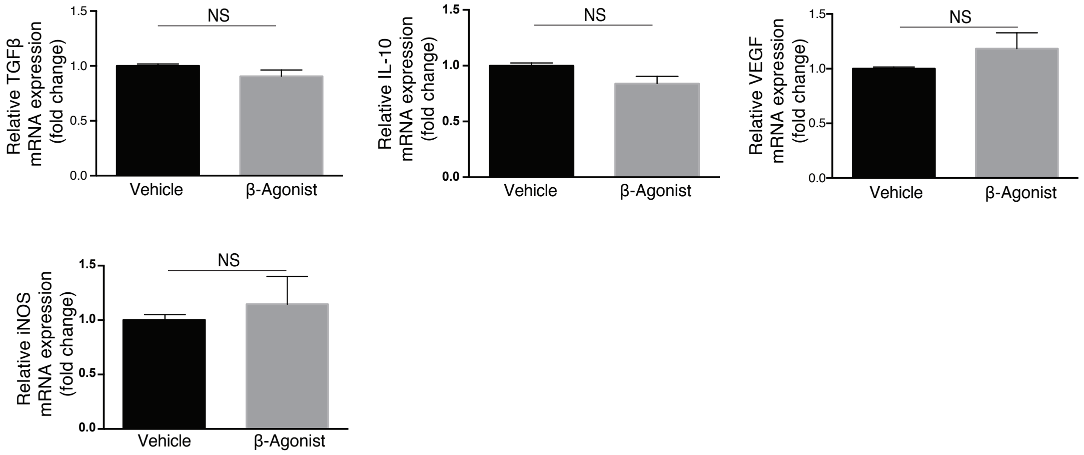

Supplementary Fig. 8

Effects of β-adrenergic agonist on bone marrow MDSCs mRNA expression of TGFβ, IL-10, VEGF, and iNOS. qPCR analysis of TGFβ, IL-10, VEGF and iNOS mRNA expression levels by MDSCs (Gr-1$^+$CD11b$^+$) sorted from the bone marrow of tumor-bearing mice, and incubated *in-vitro* with the β-adrenergic agonist (isoproterenol 1uM; TGFβ $P<0.22$, IL-10 $P<0.09$, VEGF $P<0.66$, iNOS $P<0.94$; *Mann-Whitney test* to account for the multiple comparisons; NS- not significant; n=5). *P<0.1.

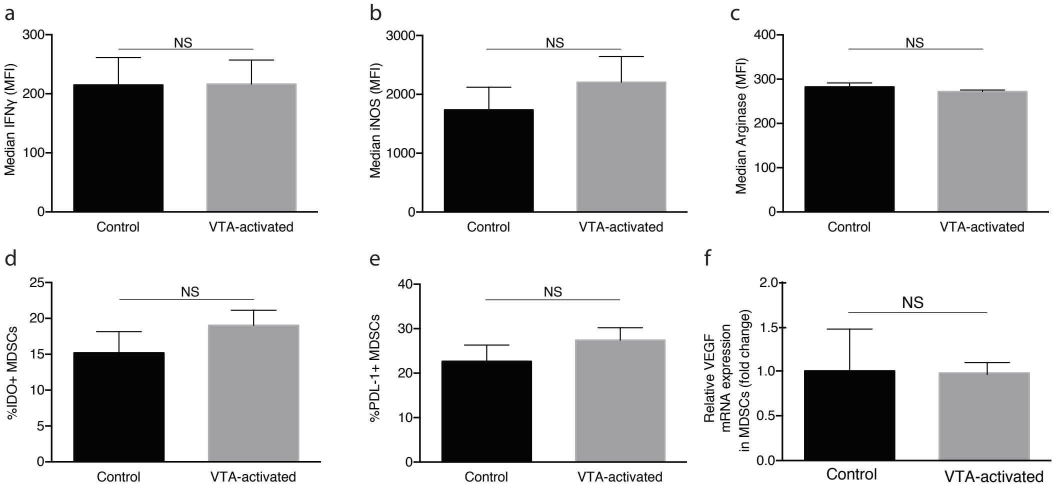

Supplementary Fig. 9

Characterization of MDSCs isolated from VTA-activated and control mice. Tumor MDSCs expression of (a) IFNγ, (b) iNOS, (c) Arginase, (d) IDO-1 and (e) PDL-1 from VTA-activated mice and their controls (injected with a control virus and treated with CNO) using flow cytometry. For IFNγ, iNOS and Arginase, the data represent mean ± s.e.m of median marker expression among tumor MDSCs from VTA-activated mice and their controls. For IDO-1 and PD-1, the data represent mean ± s.e.m of the abundance of marker-positive MDSCs from VTA-activated mice and their controls. (f) mRNA levels of VEGF expressed by MDSCs from VTA activated mice and their controls were analyzed by qPCR. (IFNγ analysis $p<0.98$ and n=8; iNOS analysis $p<0.44$ and n=8,10; for IDO-1 $p<0.35$ and n=5,4; for PD-1 $p<0.36$ and n=5,4; for Arginase $p<0.34$ and n=5,4; VEGF analysis $p<0.95$ and n=4,7; Student's *t-test*; NS-not significant).

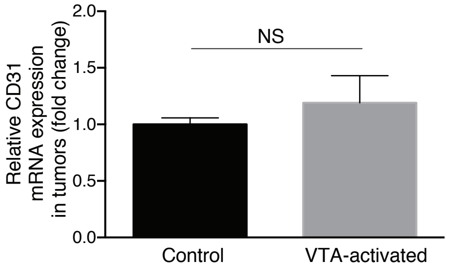

Supplementary Fig. 10

VTA activation does not affect tumor mRNA expression of CD31. Tumors from VTA-activated mice and their controls were analyzed for CD31 levels by qPCR ($p<0.41$; Student's *t-test*; NS-not significant; n=4,3).

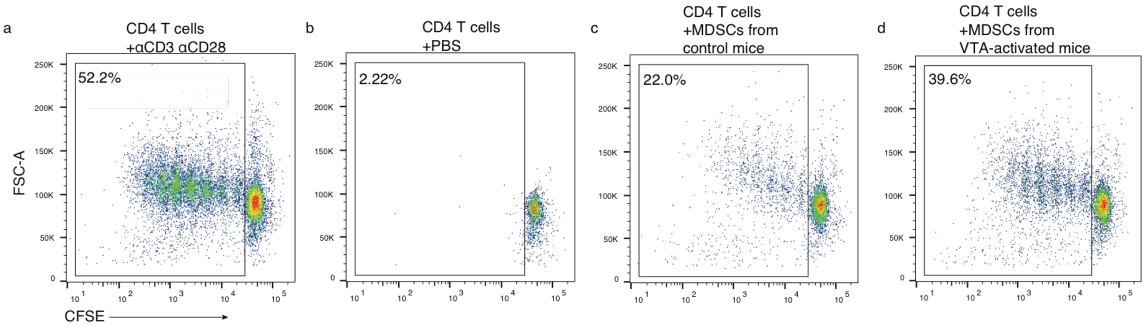

Supplementary Fig. 11

Representative dot-plots for MDSCs-suppression assay. CFSE labelled CD4 T cells were incubated under the following conditions: (a) Stimulated with anti CD3 and anti CD28 in culture medium, (b) incubated in culture medium in the absence of stimulating signals, (c) stimulated with anti CD3 and anti CD28 in the presence of MDSCs isolated from sham-virus injected mice, (d) stimulated with anti CD3 and anti CD28 in the presence of MDSCs isolated from VTA-activated mice. Cells were analyzed after 96hr incubation. The percent suppression of proliferating T cells was calculated as described in the methods.

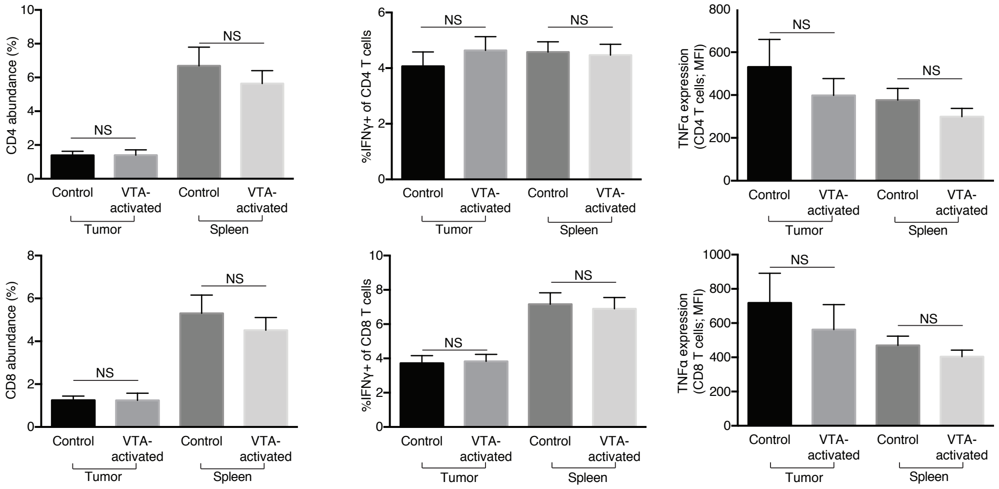

Supplementary Fig. 12

VTA activation does not affect tumor and spleen CD4 and CD8 cell abundance, nor IFNγ and TNFα expression. Flow cytometry analysis of tumor and spleen CD4 (TCRβ$^+$ CD4$^+$) and CD8 T cell (TCRβ$^+$ CD8$^+$ CD49b$^-$) abundance, and expression of IFNγ and TNFα following repeated VTA-activation. (CD4 tumor cells abundance (*P<0.98*), IFNγ$^+$ percentage (*P<0.44*), TNFα expression (*P<0.39*), n=7; spleen CD4 cells abundance (*P<0.46*), IFNγ$^+$ percentage (*P<0.84*), TNFα expression (*P<0.29*), n= 8, 7; CD8 tumor cells abundance (*P<0.99*), IFNγ$^+$ percentage (*P<0.85*) and TNFα expression (*P<0.50*), n=7; spleen CD8 cells abundance (*P<0.47*), IFNγ$^+$ percentage (*P<0.78*) and TNFα expression (*P<0.37*), n= 8, 7; Student's *t-test*; mean ± s.e.m; NS- not significant).

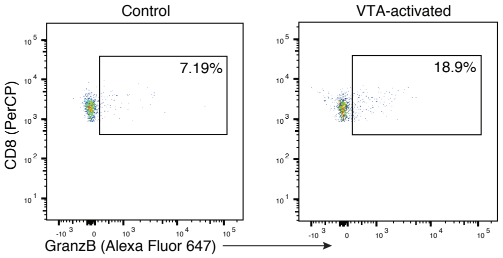

Supplementary Fig. 13

Representative dot plots of Granzyme B expression by tumor CD8 T cells from VTA activated and control mice. Representative dot plots show gated tumor CD8 T cells identified by TCRβ^{+} CD8^{+} CD49b^{-}. X axis represents Granzyme B expression, while the Y axis represents CD8 expression.

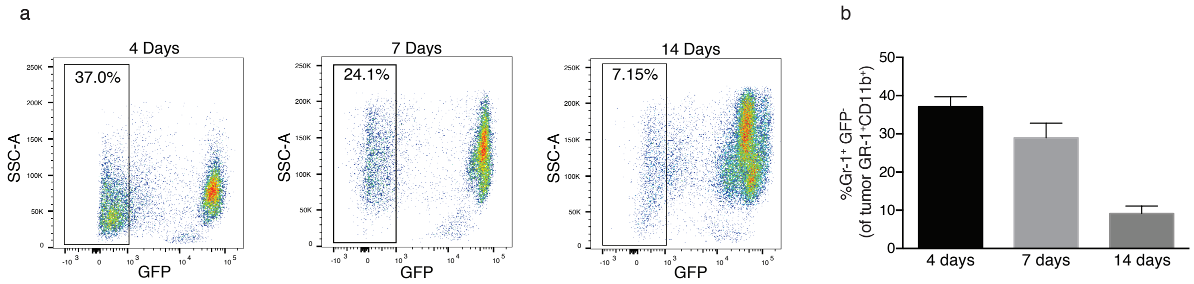

Supplementary Fig. 14

Evaluation of transferred MDSCs survival in tumor-bearing GFP mice. transferred from tumor-bearing mice into GFP mice, along with LLC tumor cells. (a) Representative images and (b) Quantification of transferred MDSCs (identified as GFP^{-} CD11b^{+} Gr-1^{+}). Their abundance was analyzed at 4,7 and 14 days after transfer out of total CD11b^{+} Gr-1^{+} cells in tumor (for 4 days n=4; for 7 days n=4; for 14 days n=2; Student's *t-test*; mean ± s.e.m).

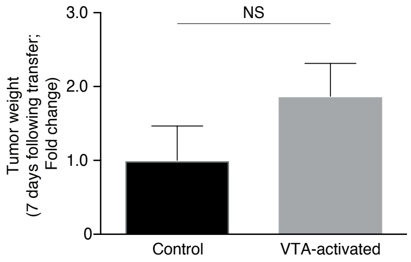

Supplementary Fig. 15

Transfer of MDSCs from VTA-activated mice and their controls along with naïve tumor cells does not affect tumor weight 7 days following transfer. MDSCs were isolated from VTA-activated mice and their controls. The cells were then co-injected along with new tumor cells into naïve recipient mice. The recipient mice were injected with an equal number of MDSCs and LLC cells. To ensure that there is a detectable tumor at this early time point, we injected a larger number of MDSCs and tumor cells, preserving the original ratio between the two cell types ($1*10^6$ of each cell type). Data is shown as fold change relative to the average of the control group ($P<0.24$; Student's *t-test*; mean ± s.e.m; NS-not significant; n=3, 4).
